# Attitudinal Profiles Toward Medical Mediation Among Healthcare Professionals: Evidence from a Scenario-Based Survey and Latent Class Analysis

**DOI:** 10.3390/healthcare14060710

**Published:** 2026-03-11

**Authors:** Olympia Lioupi, Polychronis Kostoulas, Konstadina Griva, Charalambos Billinis, Costas Tsiamis

**Affiliations:** 1 Faculty of Public and One Health, School of Health Sciences, University of Thessaly, 43100 Karditsa, Greece; pkost@uth.gr (P.K.); ktsiamis@uth.gr (C.T.); 2 Lee Kong Chian School of Medicine, Nanyang Technological University, Singapore 639798, Singapore; konstadina.griva@ntu.edu.sg; 3 Faculty of Veterinary Medicine, School of Health Sciences, University of Thessaly, 43100 Karditsa, Greece; billinis@uth.gr

**Keywords:** medical mediation, healthcare conflict, clinical ethics, scenario-based survey, latent class analysis

## Abstract

**Highlights:**

**What are the main findings?**

- Greek healthcare professionals show consistently high support for medical mediation across ethically complex scenarios, particularly in medical error disclosure.
- Latent class analysis identified three distinct attitudinal profiles, with nearly three-quarters of respondents strongly supportive despite minimal formal training.

**What are the implications of the main findings?**

- There is a clear mismatch between strong professional support for medical mediation and its limited institutionalization and related training in the Greek healthcare system.
- The findings provide empirical justification for integrating structured medical mediation training and services into healthcare policy and clinical ethics frameworks.

**Abstract:**

Medical mediation (MM) is a collaborative tool for resolving ethically complex disputes in healthcare. **Background/Objectives**: Though widely recognized in international clinical ethics, it has only been recently introduced in Greece. The objective of this study was (i) to quantify agreement with MM across three clinical scenarios, (ii) to estimate the proportion of professionals that support mediation and institutional training, and (iii) to identify distinct attitudinal profiles using latent class analysis (LCA). **Methods**: A structured, cross-sectional online questionnaire was completed by 431 healthcare professionals across Greece. The survey included three clinical vignettes (on (1) end-of-life care, (2) religious refusal of treatment, and (3) medical error disclosure), Likert-scale items on attitudes toward mediation, and demographic information. LCA was used to identify patterns of response across the scenarios and differentiate between strongly supportive, moderately supportive, and cautiously positive professional profiles. **Results**: Participants expressed strong support for mediation across all scenarios (median scores ≥ 9), with the highest support for medical error disclosure (mean 8.67 ± 2.10 and a median of 10). Most participants (97.2%, n = 419) considered mediation at least sometimes effective, and 80.7% (n = 348) endorsed institutional training. However, only 3.0% (n = 13) reported formal training and 1.9% (n = 8) reported being very familiar with MM. LCA revealed three distinct respondent profiles: strongly supportive (73.3%, n = 316), moderately supportive (14.6%, n = 63), and cautiously positive (12.1%, n = 52). Significant trends were observed across profiles for the perceived effectiveness of mediation and support for institutional training (*p* < 0.01). However, formal training and familiarity with mediation among the participants were low (<5%). **Conclusions**: Despite limited training and formal implementation, Greek healthcare professionals show high support for MM. The demand and need for structured mediation training and integration into the Greek healthcare system is strong. The identification of distinct attitudinal profiles provides insight into potential variation in organizational readiness for implementing structured mediation training

## 1. Introduction

Medical mediation (MM) is a critical tool for addressing ethical and communication challenges in modern healthcare. In settings where conflicts between patients, families, and healthcare professionals are frequent, mediation offers a structured framework for fostering dialogue, maintaining trust, and achieving ethically sustainable solutions. Globally, its effectiveness has been documented in various contexts, such as end-of-life decisions [1,2], religious objections to treatment [3], and the disclosure of medical errors [4,5]. In countries like the United States and the United Kingdom, mediation has been institutionally recognized as part of clinical ethics support services, playing a decisive role in resolving conflicts in complex clinical cases [6,7]. Further, several countries, such as Canada [8], China [9], Hong Kong [10], Italy [11], and Singapore [12], among others, have been utilizing mediation primarily in conflict resolution or end-of-life settings.

In Greece, the application of mediation in general was only recently introduced in 2010 (Law 3898/2010) on a voluntary basis and was made a mandatory pre-trial step in 2020 (Law 4640/2019) for commercial trials that cover, inter alia, medical-liability claims. Thus, the implementation of MM in Greece is currently limited. An additional constraint is that under current law it can only be applied in the case of medical errors in private healthcare settings and not in conflicts with the public health sector. Further, previous published reports from Greece are limited. The importance of nurses acting as mediators between physicians and patients in futility cases has been discussed in a recent paper [13], while an earlier report discusses the absence of mediation in transplant-related ethical disputes in Greece [14]. Since mediation has only been introduced recently, it remains largely underutilized in the healthcare sector. Nevertheless, healthcare systems in Europe and internationally are increasingly recognizing the value of structured conflict resolution mechanisms and have introduced them in institutionalized settings beyond the USA and UK [15], as is the case, for example, in China, Taiwan, and elsewhere [16]. Given the proven usefulness of MM as an alternative dispute resolution mechanism in various aspects of healthcare disputes, it is vital to investigate the perceptions and readiness of Greek healthcare professionals to adopt it.

Given the established international use of MM and its recent legislative introduction in Greece, there is a clear need to generate empirical evidence regarding healthcare professionals’ attitudes toward its potential integration into clinical practice. Knowledge of these attitudes is practically important for informing educational planning, institutional policy development, and the design of structured conflict-resolution services within healthcare settings. In particular, identifying whether support varies across ethically distinct clinical contexts and whether different attitudinal profiles exist within the professional community may provide insight into implementation readiness and potential barriers to institutional adoption. Therefore, the objectives of this cross-sectional study were the following:

(i). to quantify healthcare professionals’ agreement with the use of MM across three ethically complex clinical scenarios (end-of-life decision-making, religious refusal of treatment, and medical error disclosure);
(ii). to estimate the proportion of professionals that support the effectiveness of MM and institutional training; and
(iii). to identify distinct attitudinal profiles using latent class analysis (LCA) and examine their association with training, familiarity, and sociodemographic characteristics.

## 2. Materials and Methods

### 2.1. Survey Instrument

A structured, self-administered questionnaire was designed to assess perceptions of MM among healthcare professionals. The instrument was developed in Greek by the study team based on the published literature on MM in ethically complex clinical contexts. To ensure content clarity and face validity, the final version was reviewed by the multidisciplinary author group (public health, ethics/health policy, and clinical and legal perspectives), and minor wording refinements were made prior to dissemination. The questionnaire was pilot-tested for clarity in a small convenience group of healthcare professionals (n = 9), and minor linguistic adjustments were made. The validated questionnaire was delivered online through a secure survey platform and is available as Supplementary Materials File S1. It consisted of closed-ended items, including Likert-scale, multiple-choice, and categorical responses, and was structured into three sections.

MM was defined as a structured, facilitated dialogue process aimed at resolving healthcare-related conflicts between patients, families, and healthcare professionals. Familiarity with MM referred to self-reported awareness or understanding of the concept, measured on a five-level ordinal scale. Training on MM was categorized as no training, informal training (e.g., workshops or seminars), or formal certified training. Healthcare level referred to the respondent’s primary workplace setting and was categorized as primary care (community-based services), secondary care (district or general hospitals), or tertiary care (specialized or university hospitals).

Participation was anonymous, and no personally identifying information was collected.

The questionnaire was disseminated nationwide between January and March 2024 through professional and institutional mailing lists and online communication channels. The primary distribution route was the Athens Medical Association, the largest professional medical association in Greece, which formally disseminated the survey invitation to its registered physician members nationwide. Additional dissemination occurred through regional health networks and professional groups to ensure the inclusion of a broad spectrum of healthcare providers across levels of care.

The first section was a scenario-based assessment with three clinical vignettes, each describing a distinct situation where MM might be applicable. The three vignettes were designed to represent distinct, high-conflict clinical domains in which mediation has been described as relevant. Specifically, the scenarios were as follows:

1. Scenario 1: end-of-life decision-making. This was a case of an elderly patient in a persistent vegetative state, where a mediator facilitates consensus among family members with conflicting views, grounded in the patient’s previously expressed wishes and the clinical prognosis.
2. Scenario 2: religious refusal of treatment. A patient refused a life-saving blood transfusion due to religious beliefs. The mediator works with both the clinical team and a religious leader to identify culturally respectful and clinically acceptable alternatives.
3. Scenario 3: disclosure of medical error. In this case of an intraoperative mistake (a retained surgical sponge) the mediator supports the healthcare team in reaching an ethically sound and empathetic strategy for disclosing the error to the patient and their family.

Respondents were asked to rate their agreement with the mediator’s approach in each scenario using a 10-point Likert scale (1 = Strongly Disagree, 10 = Strongly Agree).

The second section gathered information on the attitudes, familiarity, and experience with MM and included items assessing (1) the beliefs about the effectiveness of MM in resolving healthcare conflicts, (2) the need to provide training on MM through healthcare institutions, (3) the self-reported prior involvement in a mediation process, (4) the self-reported familiarity with the concept of MM, and (5) whether the respondent had received any training in MM.

The third section was on sociodemographic and professional characteristics like age, gender, highest educational level attained, years of work experience in the health sector, current role, and level of service (e.g., primary, secondary, tertiary care).

Finally, the respondents were asked how likely they were to recommend MM to colleagues and their interest in participating in a follow-up qualitative interview (optional, with contact details).

This cross-sectional study is reported in accordance with the Strengthening the Reporting of Observational Studies in Epidemiology (STROBE) guidelines for cross-sectional studies (see Supplementary Materials File S2).

### 2.2. Inclusion and Exclusion Criteria

We used a convenience sampling approach. The survey invitation was disseminated nationwide via professional mailing lists and communication channels, with the primary distribution route being the Athens Medical Association. Inclusion criteria were as follows: (i) self-identified healthcare professional, (ii) currently practicing in Greece, and (iii) age ≥18 years. Duplicate submissions, where identifiable (e.g., identical timestamps/response patterns), and responses not meeting inclusion criteria were excluded from further analysis. The online survey platform was configured so that all key items were mandatory. Therefore, questionnaires could not be submitted with missing responses to the core study variables and only fully completed questionnaires were recorded and available for analysis.

### 2.3. Statistical Analysis

#### 2.3.1. Data Preparation

Likert-scale responses for the three clinical scenarios were treated as ordinal numeric variables (1–10). A composite score was calculated as the average of the three scenario ratings, representing overall agreement with the mediator’s approach. The remaining variables were recoded as factors where appropriate, with consistent handling of response scales.

#### 2.3.2. Sample Size and Representativeness

The final analytic sample comprised 431 fully completed questionnaires. No partially completed questionnaires were recorded due to the mandatory-response design of the survey platform. Given the exploratory nature of this cross-sectional design and the use of non-parametric and latent class analyses, a formal a priori sample-size calculation was not applicable. Instead, adequacy was judged based on established recommendations for psychometric and latent class modeling, where a minimum of 300 observations is considered sufficient for stable class identification and robust convergence in models with up to four latent profiles. The achieved sample exceeded these thresholds and ensured reliable estimation of conditional probabilities and meaningful subgroup comparisons across sociodemographic and attitudinal variables. Moreover, the respondents covered all major healthcare levels (primary, secondary, tertiary), supporting a diverse and reasonably representative profile of the Greek healthcare workforce.

#### 2.3.3. Descriptive and Exploratory Analysis

Descriptive statistics were computed for all variables with frequencies and percentages for categorical variables and mean (standard deviations) for continuous variables. Bar plots were used to visualize Likert responses that were grouped in three categories for interpretability: Disagree (1–4), Neutral (5–6), and Agree (7–10).

#### 2.3.4. Internal Consistency and Concordance

To assess the coherence of the scenario-based items, we calculated Cronbach’s alpha. Because the three vignettes intentionally represent conceptually distinct ethical contexts, alpha was interpreted as an index of coherence rather than unidimensionality. To assess whether responses significantly differed across the three scenarios, we conducted a Friedman test followed by pairwise Wilcoxon signed-rank tests with Bonferroni adjustment.

#### 2.3.5. Latent Class Analysis

To identify distinct underlying patterns of response among participants, we performed a LCA based on the ratings of the three clinical scenarios and the likelihood of recommending MM. These items were treated as categorical indicators, reflecting respondents’ agreement with the mediator’s approach in each situation.

LCA models with two to four latent classes were fitted using the poLCA package in R, with multiple random starts (nrep) to ensure convergence to a global maximum likelihood solution. Model fit was evaluated using the Bayesian Information Criterion (BIC), Akaike Information Criterion (AIC), and log-likelihood. The selected class solution was based primarily on BIC and AIC minimization and interpretability. Following the selection of the optimal number of latent classes, individuals were assigned to their most likely class (modal assignment), and class profiles were interpreted based on conditional response probabilities. A detailed description of the LCA models and fit statistics can be found in the appendix (Supplementary Materials File S3). Finally, to characterize the identified classes, we compared them on a range of attitudinal, experiential, and demographic variables, with Chi-square tests for categorical variables and Kruskal–Wallis tests or Jonckheere–Terpstra tests for ordinal variables.

All statistical tests were two-sided, and a significance level of *p* < 0.05 was considered statistically significant.

#### 2.3.6. Software

All statistical analyses were conducted using R version 4.3.2 (R Core Team, Vienna, Austria) [17] within the RStudio environment. The tidyverse suite of packages [18], including dplyr, ggplot2, and tidyr, was used for data manipulation and visualization. We used the kruskal.test() and JonckheereTerpstraTest() from the DescTools package [19] for ordinal comparisons, the psych package for reliability analysis, and the poLCA package to implement the LCA [20].

#### 2.3.7. Ethics Approval Statement

This study was a non-interventional, cross-sectional survey conducted exclusively through an anonymous, self-administered online questionnaire addressed to healthcare professionals. Participation was entirely voluntary. At the beginning of the questionnaire, participants were informed about the purpose of the study, the anonymous and confidential nature of data collection, and the use of the data solely for research purposes. No personal or sensitive identifying information was collected. Informed consent was implied by the voluntary completion of the questionnaire. The study protocol was approved by the Ethics Committee of the Faculty of Public and One Health, School of Health Sciences, University of Thessaly

## 3. Results

A total of 431 healthcare professionals completed the questionnaire. Their responses are summarized in Table 1. The gender distribution was balanced, and most respondents were aged between 41 and 60 years (59.4%). In terms of educational attainment, the majority held a bachelor’s (39.2%) or master’s degree (31.1%), while 20.9% had obtained a doctoral degree, and only 8.8% reported postdoctoral training. Nearly two-thirds (64.3%) had more than 15 years of experience in the healthcare sector, were predominantly in medical professions (93.0%), and evenly distributed across the primary (39.0%) and tertiary (43.6%) levels of care, with fewer from secondary care settings (17.4%). More than half of the respondents were not at all familiar, or reported very little familiarity, with MM, while a small proportion (1.9%) reported being very familiar with it. In line with this, training on MM was limited, with 80% reporting no training, 16.9% reporting informal training, and only 3% reporting formal training. Nevertheless, 34.1% reported that they had participated in a MM process, and support for institutional training in MM was overwhelmingly high, with 80.7% agreeing that institutions should always provide such training. Further, the perceived effectiveness of MM in resolving healthcare disputes was high, with 97.2% indicating that it was at least “sometimes” effective and 30.4% indicating it was “always” effective.

**Figure 1:**
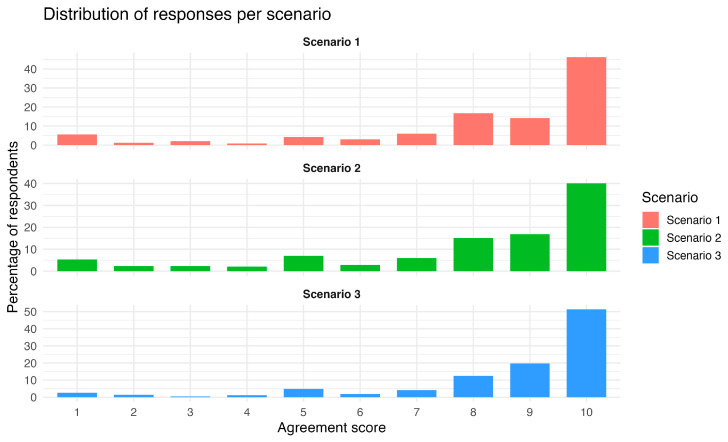
Distribution of agreement scores across clinical scenarios (Score: 1 = Strongly Disagree to 10 = Strongly Agree).

**Figure 2:**
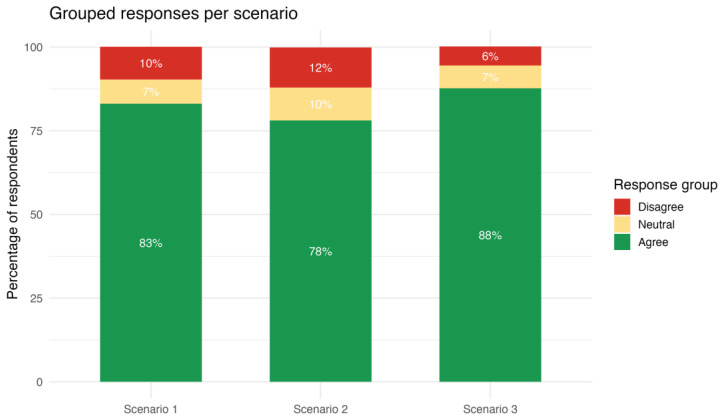
Grouped agreement levels by scenario summarizing the proportion of responses grouped as Disagree (1–4), Neutral (5–6), and Agree (7–10) for each scenario.

The participants’ ratings of the mediator’s approach generally revealed high levels of support (Figure 1 and Figure 2). The highest mean score was observed for *scenario 3* (medical error disclosure), with a mean of 8.67 (SD = 2.10) and a median of 10; *scenario 1* (end-of-life decision-making) received a mean score of 8.22 (SD = 2.52) and a median of 9, while *scenario 2* (religious refusal of treatment) had a lower mean of 7.94 (SD = 2.64) but shared the same median of 9. All scenarios had the full response range from 1 (strongly disagree) to 10 (strongly agree).

In line with these, the Friedman test for repeated measures revealed a statistically significant difference in the distribution of responses among the scenarios (χ^2^(2) = 27.54, *p* < 0.001). Pairwise comparisons using the Wilcoxon signed-rank test with Bonferroni adjustment revealed that scenario 3 (medical error disclosure) had significantly higher scores than both scenario 1 (end-of-life decision; *p* = 0.011) and scenario 2 (refusal of treatment due to religious beliefs; *p* < 0.001), while the difference between scenarios 1 and 2 was borderline significant. (*p* = 0.050).

A Cronbach’s alpha was α = 0.56 (95% CI: 0.49–0.63). Hence, there is modest internal consistency, indicating that while the three items are related, they are not highly interchangeable or reflective of a single underlying construct. Item-wise, all three scenarios showed positive item-total correlations (r = 0.74 for scen_1 and r = 0.67 for scen_3), implying that each contributes positively to the scale. Further, alpha did not increase substantially with the removal of any single item, supporting the view that the scenarios provide complementary, but not redundant, information.

**Figure 3:**
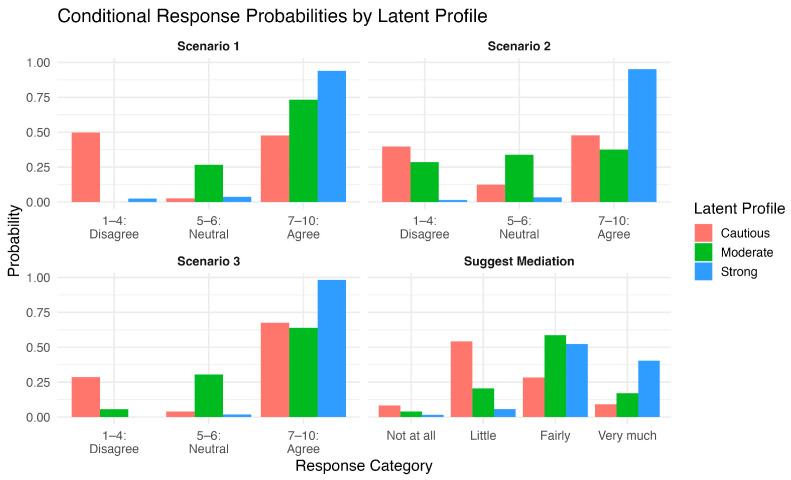
Distribution of the conditional probabilities across the three latent profiles (Cautiously positive, Moderately supportive, and Strongly supportive) by clinical scenario and likelihood of recommending MM.

LCA revealed three classes as the optimal number of distinct classes. A comparison of model fit indices for the 2–4 class solutions, including log-likelihood, AIC, BIC, and classification diagnostics, is presented in Supplementary File S3 (Table S1). Thus, three different profiles were formulated based in the ratings for the three clinical scenarios and the likelihood of recommending MM: Class 1, the “Strongly supportive” profile with participants in this class consistently rating the mediator’s approach very positively across all scenarios and having the highest probability of recommending mediation; class 2, the “Moderately supportive” profile, with members of this class expressing moderate agreement with the mediator’s role across all scenarios combined with moderate recommendation levels; and class 3, the “Cautiously positive” profile showing a mixed support of the different scenarios and their recommendation levels being lower, with a higher probability of selecting a “neutral” stance. The conditional response probabilities by class are shown in Figure 3, and the exact probabilities are provided in the supplement. The model-based proportions for each profile were as follows: 16.0% for Moderately supportive, 16.3% for Cautiously positive, and 67.6% for Strongly supportive. A posteriori modal assignment resulted in 14.6% of participants being classified as Moderately supportive (n = 63), 12.1% as Cautiously positive (n = 52), and 73.3% as Strongly supportive (n = 316).

**Figure 4:**
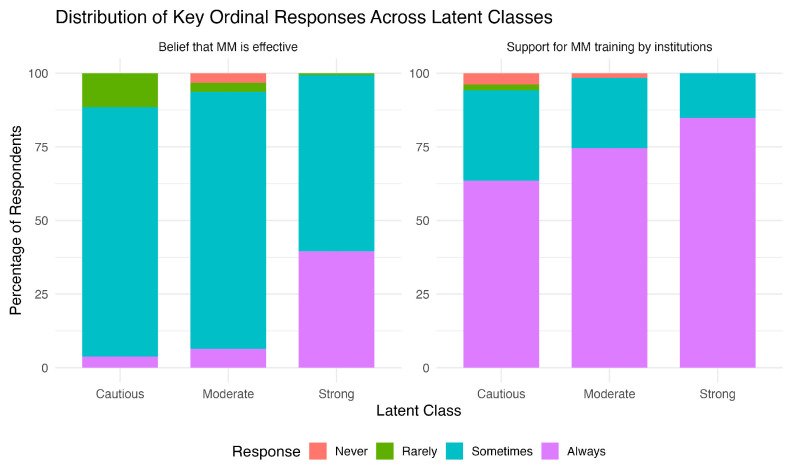
Belief that MM (MM) is effective, and the need of institutional support for MM training across the three latent profiles (Cautious, Moderate, and Strong).

Finally, the comparison of the latent classes across the attitudinal and demographic variables, via the Chi-squared and the Jonckheere–Terpstra (JT) tests, revealed that a significant trend exists for the belief that healthcare institutions should provide mediation training (trainbyinstit, JT *p* = 0.008), with recorded scores increasing significantly across profiles from cautiously positive to moderately supportive to strongly supportive. A similar trend was observed with the perceived effectiveness of mediation in healthcare (never, JT *p* < 0.001) (Figure 4). The variables of prior training, familiarity, age, sex, education, work type, and level did not reveal significant trends or differences between the three profiles.

## 4. Discussion

This is the first large-scale report on the perceptions towards MM in healthcare professionals in Greece. Our findings indicate that, despite limited formal training and familiarity with structured MM processes, Greek healthcare professionals exhibit consistently favorable attitudes toward mediation as a conflict resolution tool across a range of ethically complex clinical scenarios. The predominance of a strongly supportive profile and the significant association between more positive attitudes, perceived effectiveness of mediation, and endorsement of institutional training suggest that attitudes are shaped more by underlying beliefs and experiential value systems than by demographic characteristics or formal exposure. These results align with broader evidence suggesting that mediation fosters open communication, preserves relationships, and can serve as an effective alternative to litigation or adversarial resolution in healthcare settings [21,22].

Previous studies discussed the important role of nurses acting as mediators between physicians and patients in futility cases [13] and highlighted the lack of bioethical mediation in transplant-related ethical disputes in Greece, which is in contrast to other countries where MM has produced astounding results [14]. To the best of our knowledge, these are the only studies relating to MM in Greece. Perhaps the scarcity of relevant studies could be due to the absence of a legislative framework for mediation until recently. In comparison to other EU jurisdictions, Greece adopted mediation relatively late. Specifically, voluntary mediation was first enabled in 2010 (Law 3898/2010), followed by mandatory pre-trial mediation, which was only activated in 2020 (Law 4640/2019) and covers, inter alia, medical-liability claims. Although more than 2000 mediators have been accredited and several specialized centers exist, there are no available data on the number of conflicts that have been resolved through MM. However, the number is expected to be low given that MM under the current legislative framework can only be applied in the case of medical-liability claims with private and not public healthcare institutions. Indeed, only 3% of the participants in this study reported formal training on MM and only 1.9% reported being very familiar, while 80% reported no training at all, and more than half of the respondents were not familiar with the process. Nevertheless, at least one third of the participants reported that they had participated in an “informal” MM process during their professional life and the affirmative support for the need for institutional training in MM was overwhelmingly high, with 80.7% agreeing that institutions should always provide such training.

A notable and analytically important finding is the marked discrepancy between the high level of expressed support for MM and the very low levels of formal training and familiarity. This divergence reflects a broader recognition among healthcare professionals of the need for structured conflict-resolution mechanisms in ethically complex environments even in the absence of institutional exposure, but it may also suggest that favorable attitudes may be aspirational rather than experience-based. However, without formal training frameworks, procedural integration, and legislative clarity, supportive attitudes alone are unlikely to translate into sustainable implementation. This gap between normative endorsement and structural preparedness highlights a critical policy and organizational challenge for the institutionalization of MM in Greece.

This attitudinal pattern was further reflected in the responses to the three scenarios. Although the overall attitude of Greek Health professionals could be affected by the fact that those who chose to respond may differ systematically from those who did not, there was a consistent pattern of high agreement with the mediator’s approach across the three distinct ethically complex clinical vignettes. In all cases the median value was 9 (i.e., strong support of MM). This pattern is consistent with prior evidence indicating that mediation fosters open communication, empathy, and the preservation of relationships between patients and providers [21,23,24] It has been applied effectively across various domains, including end-of-life care [1], religious objections [3], and medical error disclosure [9], three domains that correspond to the three scenarios offered to the participants of this study. Recent systematic evidence further confirms the expanding international use of structured MM across diverse clinical conflicts, highlighting its ethical, communicative, and institutional dimensions beyond individual dispute resolution [16]. Although the responses revealed an affirmative stance on MM, they also demonstrated significant differences across the scenarios. Cronbach’s alpha indicated a modest agreement in the participants’ ratings and the response to scenario 3 (medical error disclosure) was significantly higher compared to scenarios 1 and 2; moreover, the difference between scenarios 1 and 2 was borderline significant, with scenario 2 (religious refusal of treatment) having the lowest rating. As clarified in the Methods section, the three vignettes were intentionally designed to capture conceptually distinct ethical domains. Hence, Cronbach’s alpha was interpreted as an index of coherence rather than unidimensionality. In this context, moderate internal consistency is conceptually expected and reflects the heterogeneous normative and structural constraints associated with different types of clinical conflict. This is expected, since religious objections to treatment present more complex ethical dilemmas, where mediation may face structural limitations because religious language can function as a “conversation stopper,” making compromise more challenging [3]. In contrast, medical professionals in Greece see MM as more valuable in the cases of medical errors—scenario 3—where transparency and restorative dialogue are clearly beneficial [25] and institutionalization has been promoted [4,9,26,27]. Finally, scenario 1 (end-of-life decision-making) was ranked in the middle. The use of MM in the case of end-of-life dilemmas has been proven to be a constructive way to handle emotionally charged treatment dilemmas, without disrupting the therapeutic alliance between clinicians, patients, and families [2,28,29].

Furthermore, LCA revealed three profiles of responses in terms of rating the three scenarios and the probability of suggesting mediation: the “Strongly supportive” profile, with participants consistently rating the mediator’s approach very positively across all scenarios and having the highest probability of recommending mediation, the “Moderately supportive” profile, with members of this class expressing moderate agreement with the mediator’s role across all scenarios combined with moderate recommendation levels, and the “Cautiously positive” profile, showing a mixed support of the different scenarios, having lower recommendation levels, and having a higher probability of selecting the “neutral” stance. Importantly, a posteriori modal assignment resulted in 73.3% (n = 316) of participants being classified as strongly supportive, underscoring the dominance of this attitudinal profile within the sample. Notably, there was no difference in the training and familiarity levels (because both were very low) or in the demographic characteristics (age, sex, education, work type, and level) between the profiles. This pattern is consistent with international evidence that attitudes toward conflict-resolution tools are often shaped far more by experiential and value-based factors than by sociodemographic traits. Indeed, these belief-driven differences were underscored by the significant trend we observed: from the cautiously positive to the moderately supportive to the strongly supportive profile, the proportion of respondents who (i) view mediation as an effective tool and (ii) endorse mandatory institutional training had a significant upward trend. These profiles may reflect varying degrees of organizational readiness for structured mediation implementation. The “strongly supportive” group appears aligned with institutional integration and training adoption, whereas the “moderately supportive” group may represent professionals receptive to mediation but requiring structured exposure. The “cautiously positive” profile, characterized by greater neutrality and lower recommendation probabilities, may reflect normative uncertainty or contextual reservations rather than outright resistance.

This study has limitations that should be acknowledged. First, participation was voluntary and conducted online, which may have introduced self-selection bias, as respondents more supportive of mediation could have been more likely to complete the survey. Secondly, all data were self-reported, and measures of familiarity or prior involvement in mediation processes may be affected by recall or social desirability bias. Finally, although the sample was large and disseminated nationwide, primarily through the Athens Medical Association, which is the largest medical association in Greece, some degree of geographic underrepresentation, particularly from rural or underserved regions, cannot be excluded. Nevertheless, Athens accounts for more than half of the national population and a substantial proportion of the healthcare workforce, which suggests broad coverage of the professional community. Importantly, the present study focuses on attitudinal perceptions rather than process-level implementation. It does not evaluate how MM operates within actual clinical pathways, institutional workflows, or patient outcome structures. At present, however, such structured mediation pathways are not formally embedded within the healthcare system under study. Consequently, systematic evaluation of implementation processes or downstream outcomes is not yet feasible. Future research should therefore examine mediation within formally established institutional frameworks, once such mechanisms are operational, including its potential mediating role between organizational factors and clinical outcomes. Recent empirical work in inpatient care has demonstrated how process-level variables, such as nursing actions, may mediate the relationship between medical complexity and length of stay, illustrating the value of mediation-oriented analytical frameworks in understanding healthcare outcomes [30]. At the same time, as the first nationwide study mapping healthcare professionals’ perceptions of MM in Greece, this study provides an essential foundational step for guiding such process-oriented research. Understanding prevailing attitudinal landscapes is a prerequisite for assessing institutional readiness, designing training frameworks, and embedding mediation practices within complex care systems. Despite these limitations, the consistency of responses and the clear latent class structure observed provide supportive evidence that the findings reflect meaningful patterns in attitudes toward MM among Greek healthcare professionals.

## 5. Conclusions

This work provides information on the attitude of health professionals towards MM, which has been increasingly recognized as an effective tool for conflict resolution in healthcare settings. Greek health professionals believe in the usefulness of MM and on the need of training on the field. This information can be useful for health policy makers for developing a properly structured approach to resolving disputes in healthcare.

## Figures and Tables

**Table 1:** Distribution of sociodemographic, educational, and professional characteristics of participants, and their familiarity with medical mediation (MM).

Variable	Value	Count	Percent (%)
Sex	Female	213	49.4
	Male	218	50.6
Age	20–30 years	46	10.7
	31–40 years	56	13
	41–50 years	133	30.9
	51–60 years	123	28.5
	61+ years	73	16.9
Education	Bachelor’s degree	169	39.2
	Master’s degree	134	31.1
	Doctorate (PhD)	90	20.9
	Postdoctoral studies	38	8.8
Work Experience	Less than 1 year	14	3.2
	1–5 years	44	10.2
	6–10 years	41	9.5
	11–15 years	55	12.8
	More than 15 years	277	64.3
Type of work	Administrative	18	4.2
	Allied health roles	12	2.8
	Medical professions	401	93
Healthcare level	Primary	168	39
	Secondary	75	17.4
	Tertiary	188	43.6
Familiarity with MM	Not at all	110	25.5
	Very little	129	29.9
	A little	121	28.1
	Quite a bit	63	14.6
	Very much	8	1.9
Have you participated in MM	No	284	65.9
	Yes	147	34.1
Should institutions provide training on MM?	Never	3	0.7
	Rarely	1	0.2
	Sometimes	79	18.3
	Always	348	80.7
Have you been trained on MM?	No	345	80
	Yes, informal training	73	16.9
	Yes, formal training	13	3
Do you think that MM is an effective tool for healthcare?	Never	2	0.5
	Rarely	10	2.3
	Sometimes	288	66.8
	Always	131	30.4

## Data Availability

The data presented in this study are not publicly available due to ethical and privacy restrictions, as they were collected through an anonymous survey of healthcare professionals. De-identified data may be made available from the corresponding author upon reasonable request and subject to approval by the Ethics Committee of the University of Thessaly.

## Supplementary Materials

The following supporting information can be downloaded at: https://www.mdpi.com/article/10.3390/healthcare14060710/s1, Supplementary File S1: Survey on Opinions and Experiences about Medical Mediation; Supplementary File S2: STROBE Statement—Checklist of items that should be included in reports of cross-sectional studies; Supplementary File S3: Detailed description of Latent Class Analysis Methodology and Model Diagnostics.

## Abbreviations

The following abbreviations are used in this manuscript:

MM	Medical Mediation
LCA	Latent Class Analysis
