# Supplementary material for: Attitudinal Profiles Toward Medical Mediation Among Healthcare Professionals: Evidence from a Scenario-Based Survey and Latent Class Analysis
Source: Healthcare (Basel). 2026 Mar 11;14(6):710. doi: 10.3390/healthcare14060710 (PMC13026227; doi:10.3390/healthcare14060710)
Supplement: Supplementary file 1 [file healthcare-14-00710-s001.zip › Supplementary File S1.pdf]

Survey on Opinions and Experiences about Medical Mediation

Dear participants,

we invite you to participate in a research study to help us better understand your views and experiences regarding medical mediation. Your participation in this study is voluntary, and your responses will remain confidential. The data collected will be used exclusively for research purposes and will not be shared with third parties.

Thank you for your time and contribution to this study.

Scenario 1

In a quiet hospital room, you see M., a 78-year-old woman in a vegetative state due to a severe stroke. Her two adult children are present. One, respecting their mother's previous statements, supports the withdrawal of life support. The other, driven by emotional attachment and hope, strongly opposes. A medical mediator, Dr. A., enters and facilitates a deeply emotional discussion, exploring the mother's values, the family's hopes, and the medical reality, guiding the family towards a consensus that honors M's dignity and wishes.

Do you agree or disagree with the mediator's approach to guide the family towards a consensus based on the patient's previously expressed wishes and the medical reality?

	1	2	3	4	5	6	7	8	9	10	
Strongly disagree											Strongly agree

Scenario 2

In a big city hospital, a patient refuses a life-saving blood transfusion citing religious beliefs. His doctor, Dr. N., is concerned but wants to respect his faith. L., a medical mediator specialized in cultural sensitivity, intervenes. She facilitates a dialogue between the medical team and a local priest of the specific religion, exploring alternative treatments and finding a culturally respectful and medically sound solution.

Do you agree or disagree with the mediator's approach to finding a solution that respects the patient's beliefs while also addressing medical concerns about the choice of treatment?

	1	2	3	4	5	6	7	8	9	10	
Strongly disagree											Strongly agree

Scenario 3

At a regional hospital, a surgical team led by Dr. A. realizes that a surgical sponge has been left inside a patient, Mrs. G., after the operation. As the team discusses the approach to disclosing the mistake, emotions and opinions clash. A mediator, Dr. N., guides the team through a structured discussion about ethical responsibility, patient rights, and the importance of honest communication. A unified and empathetic approach to inform Mrs. G. and her family about the mistake is decided.

Do you agree or disagree with the mediator-led strategy for transparent and empathetic communication with the patient and family after the medical error?

	1	2	3	4	5	6	7	8	9	10	
Strongly disagree											Strongly agree

Do you believe that medical mediation can be an effective tool for resolving disputes in healthcare?

☐ Always
☐ Sometimes
☐ Rarely
☐ Never

Do you believe that healthcare organizations should provide training to their employees on medical mediation?

☐ Always
☐ Sometimes
☐ Rarely
☐ Never

Have you ever been involved in a medical mediation process in healthcare?

☐ Yes
☐ No

How familiar are you with the concept of medical mediation?

☐ Not at all
☐ Minimally
☐ A little bit
☐ Enough
☐ Very

Have you received any training on medical mediation?

☐ Yes, formal training
☐ Yes, informal training
☐ No, I have not received any training

In which level of healthcare do you work?

☐ Primary care
☐ Secondary care
☐ Tertiary care
☐ Quaternary care
☐ etc. ____

What is your current professional occupation?
☐ Doctor
☐ Nurse
☐ Social Worker - Psychologist
☐ Health Sector Administration and Economics Staff
☐ etc. ______________________________

How many years of experience do you have in the healthcare sector?
☐ Less than 1 year
☐ 1-5 years
☐ 6-10 years
☐ 11-15 years
☐ Over 15 years

What is your highest level of education?
☐ Degree
☐ Postgraduate diploma
☐ Doctoral diploma, Ph.D.
☐ Post-doctoral studies

Age
☐ 20-30 years old
☐ 31-40 years old
☐ 41-50 years old
☐ 51-60 years old
☐ Over 60 years old

Gender
☐ Female
☐ Male
☐ etc. ______________________________

Country

____

How likely are you to recommend medical mediation for resolving disputes in healthcare to your colleagues?
☐ Very
☐ Enough
☐ A little bit
☐ Not at all

Would you like to share any additional thoughts or comments regarding medical mediation or your relevant experience in the healthcare sector?

____

____

If you are interested in participating in a private interview to provide more information on this topic, please share your contact information (email).

____

____
