# Supplementary material for: Attitudinal Profiles Toward Medical Mediation Among Healthcare Professionals: Evidence from a Scenario-Based Survey and Latent Class Analysis
Source: Healthcare (Basel). 2026 Mar 11;14(6):710. doi: 10.3390/healthcare14060710 (PMC13026227; doi:10.3390/healthcare14060710)
Supplement: Supplementary file 1 [file healthcare-14-00710-s001.zip › Supplementary File S3.pdf]

Supplementary File S3 - Detailed Latent Class Analysis Methodology and Model Diagnostics

S3.1 Latent Class Analysis – Statistical Framework

Latent Class Analysis (LCA) is a model-based clustering technique used to identify unobserved (latent) subgroups within a population based on patterns of responses to observed categorical indicators. LCA assumes that associations among observed variables can be explained by membership in a finite number of mutually exclusive and exhaustive latent classes. In this study, LCA was applied to identify distinct attitudinal profiles toward medical mediation among healthcare professionals.

The following variables were included as indicators:

1. scen_1: Agreement with mediation in end-of-life scenario
2. scen_2: Agreement with mediation in religious refusal scenario
3. scen_3: Agreement with mediation in medical error disclosure scenario
4. suggest: Likelihood of recommending medical mediation

The three scenario ratings (1–10 scale) were categorized into three levels, 1–4 = Disagree, 5–6 = Neutral and 7–10 = Agree. The recommendation variable ("suggest") was treated as a four-category ordinal indicator.

S3.2 Model Estimation

All analyses were conducted in R version 4.2.2 using the poLCA package. Models with 2 to 4 latent classes were estimated using maximum likelihood with multiple random starting values to reduce the risk of local maxima. Model convergence was assessed via log-likelihood stability across repetitions. Model selection was based on, log-likelihood, Akaike Information Criterion (AIC), Bayesian Information Criterion (BIC) and interpretability and theoretical coherence of classes. Because poLCA does not directly return entropy, we additionally summarized classification certainty using the average maximum posterior probability (AvePP), computed as the mean of the largest posterior class-membership probability across individuals.

S3.3 Model Fit Comparison

Table S1. Fit indices and classification summaries for competing LCA solutions

Classes	LogLik	AIC	BIC	AvePP	Min class proportion
**2**	-1154.876	2347.751	2425.007	0.900	0.269
**3**	-1143.860	2345.720	2463.637	0.857	0.160
**4**	-1133.232	2344.464	2503.042	0.869	0.042

The 3-class solution improved AIC relative to the 2-class model (2345.72 vs 2347.75) and increased log-likelihood, indicating improved fit. However, BIC favored the 2-class model. The 4-class model showed a slightly lower AIC, but substantially higher BIC and produced a very small class (minimum class proportion ≈ 4.2%), suggesting potential overfitting and limited practical utility. Fit indices jointly with interpretability and class stability suggest that the 3-class solution should be retained as the most informative representation of heterogeneity in attitudes.
